# Under 5 Energize: Tracking Progress of a Preschool Nutrition and Physical Activity Programme with Regional Measures of Body Size and Dental Health at Age of Four Years

**DOI:** 10.3390/nu9050456

**Published:** 2017-05-04

**Authors:** Elaine Rush, Vladimir Obolonkin, Leanne Young, Madeleine Kirk, Marilyn Tseng

**Affiliations:** 1 Child Health Research, Faculty of Health and Environmental Sciences, Auckland University of Technology, Auckland 0640, New Zealand; Vladimir.obolonkin@gmail.com (V.O.); Leanne.young@aut.ac.nz (L.Y.); 2 Sport Waikato, Hamilton 3240, New Zealand; madk@sportwaikato.org.nz; 3 Kinesiology Department, California Polytechnic State University, San Luis Obispo, CA 93407-0386, USA; mtseng@calpoly.edu

**Keywords:** body mass index, growth, nutrition, dental decay, early childhood education centres, indigenous children

## Abstract

To reduce weight gain and encourage healthy eating including reduced sugar intake, Under 5 Energize (U5E) was introduced to 121 early-childhood-centres in the Waikato region of New Zealand in July 2013. Using anonymized data collected from January 2013 to September 2016 through free physical assessments of all 4-year-olds provided by the NZ Ministry of Health, the prevalence of obesity and dental decay children measured in the Waikato region was examined. Data were divided into four periods representing pre-implementation and 3 years of gradual implementation. Obesity was defined according to International Obesity Task Force criteria. Of 18,774 Waikato children included in the analysis, 32% were indigenous Māori, and 32% attended an U5E centre. Pre-implementation prevalences of obesity (4%) and visible dental decay (11%) of children attending and not-attending U5E centres were not different. While obesity prevalence did not change significantly over time, prevalence of dental decay decreased among children at U5E (trend *p* = 0.003) but not non-U5E (trend *p* = 0.14) centres, such that prevalences were significantly different between children at U5E vs. non-U5E centres at Year 3 (*p* = 0.02). The U5E intervention is a small but arguably effective part of the wider system approach that is required to improve children’s future health.

## 1. Introduction

Promoting healthy eating in children establishes a foundation for healthy behaviors and good health later in life. Interventions in childcare settings have demonstrated a positive impact on nutrition [1] and weight status [2,3,4,5]. They might also be expected to have beneficial effects for dental caries because sugar intake is a shared risk factor [6,7,8], and reducing added sugar intake is a common message in such interventions. 

In New Zealand (NZ), early childcare education centres (ECECs) provide a convenient and targeted setting [9] to provide a healthy environment and to support staff and parents in contributing to the optimal health and development of young children [10]. In 2015, 96% of all children starting school had participated in an ECEC [11]. As such, ECECs also serve as intervention points to reach those most in need and thus reduce health disparities. For New Zealand children aged 2 to 14 years, for example, the prevalence of obesity is 10.8% overall but 14.8% for Māori [12] and 60% of non-Maori children were free of dental caries at age 5, compared with only 43% of Maori children [13].

This study uses national administrative data within a quasi-experimental design to evaluate the effectiveness of the Under 5 Energize (U5E) programme. The U5E programme is a tailored, healthy eating and physical activity project initiated in 2013 among 121 ECECs in the Waikato region of New Zealand in response to a request for proposals by the New Zealand Ministry of Health for services to improve maternal and child nutrition and physical activity. We expected that the programme would have measurable effects in terms of reducing prevalence of obesity and dental decay in children under 5 years of age, particularly among Maori children and among children in socioeconomically deprived areas, compared with children attending non-U5E ECECs in the same region.

## 2. Materials and Methods 

### 2.1. Setting and U5E Programme Description

The U5E programme is based on Project Energize, a region-wide, whole-school nutrition and fitness programme that has been implemented in Waikato primary schools since 2005 [14], and that has been shown to be effective in reducing accumulation of body fat in younger children, reducing rate of rise in systolic blood pressure in older children [15], and improving BMI and run speed outcomes [16]. For the U5E programme, six primary messages were emphasized: (1) more active play every day; (2) milk and water as the best choice; (3) less sweet drinks; (4) daily fruit and vegetables; (5) less energy dense snacks; and (6) less screen time.

The U5E programme was designed to reach ECECs serving underserved populations—namely Maori centers and centers in socioeconomically deprived areas. Thus, ECECs clustered in four geographical areas with higher socioeconomic deprivation (defined by the New Zealand deprivation index [17]) were purposefully selected for inclusion in the U5E programme. Of 131 ECECs invited, 121 (92%) agreed to participate.

Four ‘Under 5 Energizers’ (U5Energizers) employed by Sport Waikato and two subcontracted community health providers support the delivery and development of the programme, each working with ~30 ECECs. Sport Waikato is a community-based, not-for-profit, regional sports trust funded by Sport NZ, government, health providers, funding partners, and private organizations and bequests. U5Energizers are nurses, teachers or graduates of exercise/nutrition or physical education, with two previously working as kaiwhakahaere (Māori coordinators) with Te Kōhanga Reo, a total immersion Māori language programme for young children. From induction, the U5Energizers participate in monthly team training to share experiences, resources and skills. The philosophy adopted is that U5Energizers provide support, expertise and suggestions but are not additional teaching staff. 

Every six months for the first year (starting July 2013), and annually thereafter, a designated representative from each ECEC works with their U5Energizer to identify the ECEC’s nutrition and physical activity priorities. For each ECEC, an individualised action plan was then developed and delivered based on ECEC-specific needs. For all ECECs, the first activities after developing action plans concerned the messages that water and milk are the best choices, and that fewer sweet drinks should be consumed. Examples of specific activities used within action plans to promote these messages are shown in Table 1. Thus, for example, one immediate change following sugary drinks workshops (held at ECECs that expressed interest in such a workshop) was communication to families that water would be provided in future as the only choice, that sugary drinks brought from home would be returned home, and reasons for this. This was also adopted by educators at many ECECs as best practice modelling for children and parents. 

### 2.2. Measures

The B4 School Check (B4SC) is a free health and development assessment offered to all New Zealand children at age 4 years by the Ministry of Health [19]. B4SCs are conducted by public health nurses and include health, behavioural, and developmental questionnaires; measurement of height and weight; and hearing, vision, and oral health screens. Public health nurses conduct the B4SC following procedures detailed in the Well Child/Tamariki Ora Programme Practitioner Handbook [20] and the Healthy Smile, Healthy Child Oral Health Guide for Well Child Providers [21]. B4SC data provided as part of a formal agreement with the NZ Ministry of Health for the purposes of this study include: child’s month of birth, sex, ethnicity (recorded as Māori, Pacific or European and other), deprivation of place of child’s residence (1–5 with 5 the poorest), district health board (20 areas that cover the whole of New Zealand), date of measurement, ECEC attended, height and weight [20], a “lift the lip” assessment and whether enrolled with the dental service or not. 

Lift the lip [21] is a quick technique meant to be used by non-oral health professionals to screen for visible dental decay by looking for patches, discolorations, filled teeth and/or visible decay. Stages of visible decay are scored from 1, representing no visible decay, to 6, with stages 2–6 representing stages of increasing visible decay (Table 2) [21]. Children with any signs of visible decay, active destruction of the tooth surface, are referred to dental services for immediate treatment.

Each child living in the Waikato district health board area was classified as attending either an U5E or a non-U5E ECEC using an algorithm that looked for a 90+% match between the name of the ECEC given by the parent/caregiver and the list of U5E ECECs. The matching procedure also included cross-checking the location provided by the parent/caregiver addresses in ECEC rolls. These analyses therefore included data from 121 U5E and 425 non-U5E ECECs (as at 2013). Data from the period January 2013 to September 2016 were divided into four periods representing pre-implementation (January 2013 to September 2013), year 1 (October 2013–September 2014) year 2 (October 2014–September 2015) and year 3 (October 2015–September 2016). 

We used two variables to evaluate the effectiveness of participation in the U5E programme: (1) IOTF gradings for obesity, overweight and normal [22] and (2) a dichotomized variable representing any visible decay (representing children referred for immediate treatment) vs. no decay [21].

The AUT Ethics committee confirmed that ethics approval was not required for the analysis of this anonymised, de-identified data where only sex of the child, ethnic group, month of birth and name of ECEC attended, if any, was provided.

### 2.3. Statistical Analysis

Of 210,926 children assessed as part of the B4SC in New Zealand between January 2013 and September 2016, 19,069 were in the Waikato region. From these, we excluded 295 (including 18 Pacific children) with a WHO height, weight, or BMI z-score less than −3 or more than +4. Pacific children showed a unique pattern of obesity but were too few in number for separate analysis, so a further 728 were excluded, leaving 18,046 children.

All analyses were conducted using R version 3.0.2 [23]. Proportions were compared using chi-squared tests. Asymptotic chi squared tests for trend were applied to the four stages of data collection to determine the significance and direction of any changes of prevalence with time.

## 3. Results

Of 18,046 Waikato children included in the analysis, 32% attended an U5E centre, 64% attended a non U5E centre and 4% did not attend any ECE. Thirty-two percent were indigenous Māori. Of the 32% attending a U5E centre 52% were living in quintiles 4 and 5 of deprivation (the poorest) compared with 43% of the children not attending U5E centres. There were no significant differences in sex or ethnic group proportions between U5E and non-U5E children, or over time.

Prevalences of obesity (4%) and visible dental decay (11%) were not different between children attending vs. not-attending U5E centres assessed during pre-implementation. IOTF-defined prevalence of obesity did not change significantly over time, such that prevalence of obesity for children in U5E vs. non-U5E centres remained comparable at Year 3 (Table 3).

Over the 3 years, prevalence of dental decay decreased from 10.9% to 8.7% (trend *p* = 0.003) among children at U5E centres, but not non-U5E centres (11.4% to 10.7%, trend *p* = 0.14), resulting in a statistically significant difference between children at U5E vs. non-U5E centres at Year 3 (*p* = 0.02) (Table 1). Overall, 90% of children recorded in the B4SC that they were enrolled with a dental service; 80% of children with visible dental decay were enrolled compared to 91% without decay. Among Maori children, dental decay prevalence decreased from 19.2% at pre-implementation to 15.7% at Year 3 at U5E centres (trend *p* = 0.02), compared with a non-significant change from 21.5% to 20.2% (trend *p* = 0.31) among Maori children at non-U5E centres, resulting in a statistically significant difference between the two groups by Year 3 (8.7%:10.7% 95% CI difference 0.2, 3.7, *p* = 0.026). Prevalence of dental decay decreased significantly in non-Maori children regardless of U5E attendance, but the decline was steeper at U5E centres (decrease from 6.4% to 4.2% vs. 6.9% to 5.4% in non-U5E centres, trend *p* = 0.01 for both). The children living in the more deprived areas, quintiles 4 and 5, had higher prevalences of obesity and dental decay than overall (Table 1).

The pattern of change analysed separately by sex was similar.

## 4. Discussion

A primary finding from this study was a significant decrease in prevalence of dental decay in Waikato children attending U5E centres, compared with children attending non-U5E centres, as indicated by routinely collected administrative data among preschoolers, although a significant decrease in prevalence of obesity was not detected. The ECEC selected for the U5E programme had a higher proportion of Māori, indigenous children and those in more deprived areas. In the third year, substantially less Māori children enrolled in U5E centres had visible dental decay compared with Māori children not enrolled in U5E ECEC: 16 out of 100 compared with 20 out of 100. 

The first and an ongoing initiative of the U5E programme was the reduction or abolition of sugary drinks and added sugar in the ECEC environment and at home. These were among the primary messages of the U5E programme and the primary topics of workshops, interactive displays, activities, and policy changes in participating U5E ECECs. This reduction in sugar intake may have more immediate and greater impact on tooth decay [7] than the rate of physical growth [8].

A systematic review of primary school-based randomised controlled trials [24] included one study with caries and three studies with plaque as the primary outcome. All four studies provided evidence of a beneficial effect, although the interventions varied considerably and did not uniformly include a sugary beverage component. One community-based intervention in US Pacific Northwest American Indian communities targeted parents, the family setting, and whole communities to deliver messages encouraging breastfeeding and consumption of water for thirst and discouraging sugar-sweetened beverages for infants and toddlers; the study found improvements in all three intervention communities compared with one control community [25]. A recent study in South Auckland, New Zealand found that children attending a school with a healthy food policy had fewer caries than children attending other schools in the same region without such a policy [26]. Together, despite a dearth of evidence from randomised trials, these studies conducted in real-world settings suggest that environmental interventions targeting consumption of cariogenic foods have the potential for visible impact on oral health in children.

These data showed no significant impact on obesity prevalence, although other school-based studies have shown an effect. An effect of U5E may be diluted because of the effects of our primary school programme Energize, in all Waikato primary schools, which are attended by older siblings of young children. Project Energize has been active in Waikato schools since 2005 and has been shown to be effective in improving fitness and reducing BMI [15,16]. Project Energize effects may filter out to other children in the Waikato, for example via siblings, family, friends and community activities, through what has been described by others [27] as “a viral-like spread of obesity prevention efforts”.

A limitation of this analysis is that estimated prevalence of dental decay depended on a quick, non-calibrated oral health screen by non-oral health professionals rather than a full dental examination. Our estimates also focused on active, visible decay including visible caries and filled teeth. As such, a strength of the measure is that it represents an actionable measure reflecting the proportion of children needing referral for immediate treatment. In the two groups of children attending and not attending U5E centres, measurement and random and systematic errors of the lift the lip screen can be assumed to be the same as the screening was independent of child care centre. Another limitation is that children’s length of attendance at their reported ECEC at the time of their B4SC was not known and could have varied substantially. In addition, because each U5E ECEC has its own action plan, ‘dose’ of delivery of the programme varies. These results serve to provide preliminary evidence of the impact of the U5E programme among the ECECs that adopted the programme, even despite the limitations of using administrative data. Given the implications for the health of an underserved population, our findings require confirmation with further monitoring of children in the region.

A strength of this study is the large sample size; almost 90% [19] of the population and 83% of Māori participate in the B4SC, which is voluntary but free. These findings indicate the utility of using routinely collected national data for the purpose of evaluating the impact of programmes in a real-world setting.

Our results extend the findings of a small but growing body of evidence suggesting that programmes aimed at reducing sugary beverage consumption and encouraging milk and water consumption have a beneficial effect on oral health. Using nationally collected administrative data, our analysis is the largest to demonstrate an effect on dental decay comparing 6090 children in 121 ECECs implementing the U5E programme to 12,684 children in 425 centres without the programme. Our results suggest that the U5E intervention is a small but arguably effective part of the system approach that is required to improve health for children in the future.

## Figures and Tables

**Table 1:** Examples of activities and common topics.

Examples of Activities	Common Topics	
1. Workshops with educators and parents	- Sugary drinks	
	- Healthy lunchboxes	
	- Nutrition label reading	
2. Modeling and similar in-class activities	- Promoting water as the only drink offered	
3. Interactive displays for parents and children	- Oral health	
	- Healthy lunchboxes	
	- Nutrition label reading	
4. Distribution of regular newsletters and other printed resources to highlight messages and provide practical tips	- Sugar content of commonly consumed drinks shown as teaspoons of sugar	
	- New programme activities and resources	
		- Ideas to promote healthy eating and physical activity at special school and culural events, such as birthdays and Easter
5. Guidance on ECEC-specific issues	- Menu planning	
	- Developing lunchbox and snack guidelines	
	- Providing water for morning tea and not sugary beverages	
	- Setting expectations for birthday celebrations	
	- Communicating with parents about new nutrition policies	
		- Working towards New Zealand Heart Foundation’s Healthy Heart Awards [18] recognizing ECEC achievements in creating healthy eating and physical activity environments

ECEC Early childhood education centres.

**Table 2:** Lift the lip ^1^ scoring for progression of decay.

Score	Progression of Decay
1	Healthy teeth and gums. No signs of decay and only a little plaque
2	Chalky patches and enamel breakdown on the side of the front teeth.
3	Clearly visible decayed front teeth, both in-between upper front teeth, and along the gumline
4	Well-advanced decay. The crowns of the top teeth are breaking down and decay is starting between the bottom teeth
5	Only the roots of the top teeth are left.
6	Deep decay in the lower back teeth (molars).

^1^ [21].

**Table 3:** Prevalence of obesity and prevalence of any progression to decay by stage and factor.

Measure	Energize				Non-Energize			
Year	Pre	1	2	3	Pre	1	2	3
N	1189	1657	1584	1660	2448	3404	3422	3410
^†^ Non-Maori (%, *n*)	63.8 (760)	64.2 (1061)	60.6 (958)	61.3 (1018)	69.6 (1702)	68.5 (2331)	66.3 (2268)	65.8 (2244)
Maori (%, *n*)	31.7 (375)	31.0 (516)	34.2 (544)	34.0 (564)	26.9 (661)	28.9 (984)	30.1 (1031)	30.3 (1033)
^††^ Deprivation 4,5 (%, *n*)	52.3 (622)	53.6 (888)	52.0 (824)	55.6 (923)	42.5 (1041)	42.6 (1451)	41.7 (1428)	47.5 (1619)
Obesity (%)								
All children	4.3	4.4	4.3	3.7	4.1	3.6	4.3	4.4
Non-Maori	3.7	2.6	3.5	2.6	2.5	2.1	2.5	3.0
Maori	5.3	7.6	4.6	5.7	7.2	6.7	7.4	6.4
Deprivation 4,5	4.2	5.1	5.0	4.8	5. 7	5.0	5.7	5.9
Any Dental decay (%)								
All children	10.9	12.5	9.8	8.7 *	11.4	12.0	10.6	10.7
Non-Maori	6.4	7.7	6.3	4.2 *	6.9	8.0	6.3	5.4 *
Maori	19.2	21.6	14.4	15.7 *	21.5	20.9	18.1	20.2
Deprivation 4,5	12.1	16.3	12.9	11.9	15.8	16.0	15.6	15.8

^†^ Non-Maori excludes Pacific; ^††^ deprivation quintiles 4 and 5 are most deprived; * *p* < 0.05 chi squared asymptotic test for downward trend from pre-implementation to year 3.

## References

[1] Iaia M., Pasini M., Burnazzi A., Vitali P., Allara E., Farneti M. (2017). An educational intervention to promote healthy lifestyles in preschool children: A cluster-RCT. Int. J. Obes. (Lond.).

[2] Bluford D.A., Sherry B., Scanlon K.S. (2007). Interventions to prevent or treat obesity in preschool children: A review of evaluated programs. Obesity (Silver Spring).

[3] Waters E., de Silva-Sanigorski A., Hall B.J., Brown T., Campbell K.J., Gao Y., Armstrong R., Prosser L., Summerbell C.D. (2005). Interventions for preventing obesity in children. Cochrane Database Syst. Rev..

[4] Zhou Y.E., Emerson J.S., Levine R.S., Kihlberg C.J., Hull P.C. (2014). Childhood obesity prevention interventions in childcare settings: Systematic review of randomized and nonrandomized controlled trials. Am. J. Health Promot..

[5] Sisson S.B., Krampe M., Anundson K., Castle S. (2016). Obesity prevention and obesogenic behavior interventions in child care: A systematic review. Prev. Med..

[6] Moynihan P.J., Kelly S.A. (2014). Effect on caries of restricting sugars intake: Systematic review to inform WHO guidelines. J. Dent. Res..

[7] Moynihan P. (2016). Sugars and Dental Caries: Evidence for Setting a Recommended Threshold for Intake. Adv. Nutr..

[8] Te Morenga L., Mallard S., Mann J. (2013). Dietary sugars and body weight: Systematic review and meta-analyses of randomised controlled trials and cohort studies. BMJ.

[9] Larson N., Ward D.S., Neelon S.B., Story M. (2011). What role can child-care settings play in obesity prevention? A review of the evidence and call for research efforts. J. Am. Diet. Assoc..

[10] Robinson S., Yardy K., Carter V. (2012). A narrative literature review of the development of obesity in infancy and childhood. J. Child Health Care.

[11] Ministry of Education Participation in Early Childhood Education. http://www.educationcounts.govt.nz/statistics/ece2/ece-indicators/1923.

[12] Ministry of Health (2015). Annual Update of Key Results 2014/15: New Zealand Health Survey.

[13] Ministry of Health (2015). Tatau Kahukura: Māori Health Chart Book 2015.

[14] Rush E., Cairncross C., Williams M.H., Tseng M., Coppinger T., McLennan S., Latimer K. (2016). Project Energize: Intervention development and 10 years of progress in preventing childhood obesity. BMC Res. Notes.

[15] Rush E., Reed P., McLennan S., Coppinger T., Simmons D., Graham D. (2012). A school-based obesity control programme: Project Energize. Two-year outcomes. Br. J. Nutr..

[16] Rush E., McLennan S., Obolonkin V., Vandal A.C., Hamlin M., Simmons D., Graham D. (2014). Project Energize: Whole-region primary school nutrition and physical activity programme; Evaluation of body size and fitness 5 years after the randomised controlled trial. Br. J. Nutr..

[17] Salmond C., Crampton P. (2002). NZDep2001 Index of Deprivation.

[18] Heart Foundation Healthy Heart Award. http://www.learnbyheart.org.nz/index.php/ece/healthy-heart-award.

[19] Ministry of Health B4 School Check Information for the Health Sector. http://www.health.govt.nz/our-work/life-stages/child-health/b4-school-check/b4-school-check-information-health-sector.

[20] Ministry of Health Well Child/Tamariki Ora Programme Practitioner Handbook: Supporting Families and Whānau to Promote Their Child’s Health and Development Revised 2014. http://www.health.govt.nz/publication/well-child-tamariki-ora-programme-practitioner-handbook-2013.

[21] New Zealand Dental Association Healthy Smile, Healthy Child. http://www.healthysmiles.org.nz/assets/pdf/HealthySmilesBooklet-3rdEdition_sml.pdf.

[22] Cole T.J., Lobstein T. (2012). Extended international (IOTF) body mass index cut-offs for thinness, overweight and obesity. Pediatr. Obes..

[23] R Development Core Team (2013). R: A Language and Environment for Statistical Computing.

[24] Cooper A.M., O’Malley L.A., Elison S.N., Armstrong R., Burnside G., Adair P., Dugdill L., Pine C. (2013). Primary school-based behavioural interventions for preventing caries. Cochrane Database Syst. Rev..

[25] Maupome G., Karanja N., Ritenbaugh C., Lutz T., Aickin M., Becker T. (2010). Dental caries in American Indian toddlers after a community-based beverage intervention. Ethn. Dis..

[26] Thornley S., Marshall R., Reynolds G., Koopu P., Sundborn G., Schofield G. (2017). Low sugar nutrition policies and dental caries: A study of primary schools in South Auckland. J. Paediatr. Child Health.

[27] Sanigorski A.M., Bell A.C., Kremer P.J., Cuttler R., Swinburn B.A. (2008). Reducing unhealthy weight gain in children through community capacity-building: Results of a quasi-experimental intervention program, Be Active Eat Well. Int. J. Obes. (Lond.).

